# Poly Implant Prothèse silicone breast implants: implant dynamics and capsular contracture

**DOI:** 10.1007/s00238-018-1427-y

**Published:** 2018-06-01

**Authors:** Y. Bachour, Z. C. M. Heinze, T. S. Dormaar, W. G. van Selms, M. J. P. F. Ritt, F. B. Niessen

**Affiliations:** 1 Department of Plastic, Reconstructive and Hand Surgery, VU University Medical Center, De Boelelaan 1117, PO Box 7057, 1007 MB Amsterdam, The Netherlands; 2 Department of Plastic, Reconstructive and Hand Surgery, OLVG location West, 1061 AE Amsterdam, The Netherlands

**Keywords:** Poly Implant Prothèse, PIP, Implant dynamics, Capsular contracture

## Abstract

**Background:**

The Poly Implant Prothèse (PIP) implants were withdrawn from the market in 2010 due to the use of low-grade silicone, causing a high risk for implant rupture. The aim of this study was to investigate the implant dynamics of PIP breast implants, as well as to determine the rate and predictors of implant gel bleeding, rupture, and capsular contracture in PIP implants.

**Methods:**

Eighty women with a total of 152 PIP implants who underwent a reoperation in 2012 were enrolled in this study. Physical investigation included assessing the Baker score and demographics were retrospectively traced in medical records. The pre- and post-operative volumes of the implants were calculated and their state was determined intraoperatively by the surgeon.

**Results:**

The implants were removed after a mean implant duration of 11 ± 2.1 years. Gel bleed and implant rupture occurred in respectively 42 and 25% of the implants. Intact implants had post-operative volume increase as well as decrease. There was a correlation between gel bleeding and more post-operative implant volume increase (*P* ≤ 0.05). Capsular contracture had a protective effect against post-operative implant volume increase (*P* ≤ 0.05), while a post-operative implant volume increase provided a protective influence in developing capsular contracture (*P* ≤ 0.05). Additionally, implant rupture led to a higher risk of capsular contracture (*P* ≤ 0.05).

**Conclusions:**

We managed to illustrate that PIP implant shells were too permeable and that there is a correlation between gel bleeding and the increase of the post-operative implant volume. Implant rupture led to a higher risk for developing capsular contracture.

Level of evidence: Level III, risk / prognostic study.

**Electronic supplementary material:**

The online version of this article (10.1007/s00238-018-1427-y) contains supplementary material, which is available to authorized users.

## Introduction

In 2007, the quality of the silicone breast implants manufactured by Poly Implant Prothèse (PIP) was put into question, as physicians started reporting higher rupture rates among patients that were treated with PIP branded implants [1, 2]. It was not until 2010 that the French Medical Regulatory Authority (ANSM) declared that silicone breast implants manufactured by PIP were illegally authorized [3]. As a consequence, medical use of the PIP implants was prohibited in several European countries, including the Netherlands. Moreover, the Dutch Health Care Inspectorate (IGZ) recommended institutions to have these implants removed from the patients.

Several studies have reported on the chemical and physiochemical properties of PIP implants. The ANSM found that the implants contained low-grade silicone gel and shells, so patients who underwent breast surgery using PIP implants in the past were at a higher risk of an implant shell rupture [3]. Additional tests conducted in 2012 by a British NHS Expert Group determined high levels of low molecular weight cyclic siloxanes (D4–6) in PIP silicone gel, but found that these siloxanes did not have cytotoxic or genotoxic effects on the body [4]. Other studies demonstrated that the shell barrier which should have prevented the implant from bleeding was missing [5, 6]. However, the full effect of the chemical and physiochemical properties of implants on the implant dynamics (e.g., shell permeability, gel bleed, and gel rupture) is unknown.

Capsular contracture is the most common complication after breast implant implantation [7–10]. It occurs in 4–17% of esthetic or reconstructive patients that have undergone breast surgery [11]. Symptoms include hardening, pain, and deformation of the breast. As a tool to assess the grade of capsular contracture after palpating the breast, clinicians use the Baker score, illustrated in Table 1 [12]. It is unclear what exactly causes capsular contracture; some hypothesize that a biofilm created after a subclinical bacterial infection stimulates this contraction [13–16], while others think an immunologic response is the source [17, 18]. The leakage of silicone gel has also been associated with contraction of the capsule, whether through rupture or gel bleeding [19].

**Table 1:** Baker score of severity of capsular contraction

Baker score	Symptoms and signs
1	Non-palpable capsule and soft
2	Minimal hardness, not visible
3	Moderate hardness, visible disfiguring
4	Hard, painful, cold breasts

To date, few studies have studied the implant dynamics and its relationship with capsular contracture. Therefore, the primary purpose of this study was to assess the implant dynamics of PIP breast implants and the risk factors related to these implant dynamics. The second purpose of this study was to analyze the rate and predictors for developing capsular contracture in PIP implants.

## Materials and methods

### Subjects

In 2012, patients that underwent breast augmentation or reconstruction with PIP implants at the OLVG Hospital in Amsterdam, the Netherlands, were recalled to the clinic for a reoperation to remove or replace their implants. The measurements of 102 patients with 194 breast augmentations and reconstructions were reported. This study includes a consecutive cohort of patients that underwent a reoperation between March 2012 and September 2012 for removal or replacement of their breast implants.

Patients who received breast implants other than PIP were excluded (*n* = 35 breasts), as they were reoperated for other indications than high risk of rupture. For our analysis, we also excluded patients that primarily underwent breast reconstruction, as they constituted a minority within the sample (*n* = 7) and tended to have a higher chance of developing complications compared to breast augmentations [20]. The final cohort comprised of 80 patients with 152 breast reoperations.

All the women who were recalled were first seen in the outpatient clinic, where they underwent physical examination of the breast and axillae. Each breast was inspected and palpated and possible axillary lymphomas were noted. The four-grade Baker score was then determined for the first time. In some cases, the physician (re-) assessed the Baker score at the operation room. In the cases where the Baker score was determined at the two aforementioned moments, the most recent score was considered as the final Baker score. A clinical Baker score of 3 or 4 was further considered as capsular contracture in this study.

Patient medical records were manually reviewed for patient demographics, surgery-related information, and implant characteristics.

### Implant characteristics

As soon as the implants were surgically removed, their integrity was recorded either as intact, gel bleeding, or ruptured. Implants were considered intact when no tears were found in the shell. Gel bleeding was reported when the shell was intact but a surrounding substance that stuck to the glove of the surgeon was apparent. Ruptured implants had holes or tears in the shell. All implants were immediately put on a digital weighing scale providing the post-operative mass (in grams (gr)). When the implants were ruptured, as much as possible of the implant substance was measured. This measuring procedure was attempted in a similar step-by-step manner by briefed theater nurses. When silicone lymphomas (siliconomas) were found during the operation, they were removed by the surgeon and sent for pathologic evaluation.

### Implant volume versus mass

All the breast implants display their (pre-operative) volume information on the package, rather than the mass. As we did not know the pre-operative mass of the implants and could not contact the manufacturer of PIP in order to know the density of the silicone, we measured the density of an unused PIP implant with the help of the Dutch National Institute for Public Health and the Environment (RIVM). Using the water displacement method with measuring containers of adequate circumference, the density was found to be 0.975 g/ml, which we used to convert the post-operative mass to volume. This converted volume was used in the analyses.

### Statistical analysis

The object of investigation was both the woman and the breast. Statistical analysis was done per breast. As most patients were operated on both breasts, the usual random effect that an unselected group of individual study objects should provide was not achieved as when using the breasts for analyses. We tested this with the use of Intra-class Coefficient Correlation tests. In order to correct this variance, multi-leveled tests were used for univariate as well as for multivariate analyses. For the nominal outcomes, a generalized linear mixed model test was used. For the dichotomous outcomes, we used the generalized estimating equations (GEE) test. Multivariable logistic regression analysis was done for all clinically relevant factors, as illustrated in the respective tables. We corrected for variables which could be possible confounders. These were the patient-related characteristics: age, body mass index, alcohol drinking, and years between operations. As, overall, this did not lead to relevant changes in the results, these outcomes are not portrayed in the tables.

Results were reported as odds ratios (OR) with 95% confidence intervals (CI) and *P* values. A two-tailed *P* value of less than 0.05 was considered statistically significant. All statistical analyses were performed using SPSS® 22 (SPSS, Inc., Illinois, USA).

## Results

The mean age of the 80 patients at the time of explantation was 53 ± 12 years. The mean body mass index was 24 ± 4.5 kg/m^2^ which falls under the category of normal weight. There were 41% smokers and 58% reported alcohol intake. Breast sides were almost equally divided, with 51% having had a breast implant on the left side of the body. The bra size could not be used for further analyses as there was not enough variable data for the tests. Breast implants were implanted for a mean of 11 ± 2.1 years. The majority of the patients did not have capsular contracture. Baker scores were 1 in 58%, 2 in 28%, 3 in 8.3%, and 4 in 5.3%. Table 2 summarizes these patient characteristics. There were two patients that developed siliconomas, which was too small a number to use in further analyses.

**Table 2:** Patient characteristics (*n* = 152)

	Mean (SD*)
Age (years)	53 (12)
	(*n* = 144)
Body mass index (kg/m^2^)	24 (4.5)
	(*n* = 132)
	*n* (%)
Smoker	52 (41%)
	(*n* = 137)
Alcohol drinker	80 (58%)
	(*n* = 152)
Breast side: left	77 (51%)
	Mean (SD*)
Years between operations	11 (2.1)
	(*n* = 132)
Baker score:	*n* (%)
Score 1	77 (58%)
Score 2	37 (28%)
Score 3	11 (8.3%)
Score 4	7 (5.3%)

*SD standard deviation

The PIP implants were all round and textured. Post-operative measurements showed a decrease in volume in 19% of the implants and an increase in 62%. In 19% of the implants, there was no change in the post-operative volume. There was a well-divided group regarding post-operative implant state, with 33% intact, 42% with gel bleeding, and 25% ruptured (Table 3). Of the 18 breasts that developed capsular contracture, 39% had intact implants, 22% gel bleed, and 39% ruptured.

**Table 3:** Breast implant characteristics (*n* = 152)

	*n* (%)
Implant volume post-operatively:	(*n* = 139)
No change	26 (19%)
Decrease	26 (19%)
Increase	87 (62%)
Implant state post-operatively:	(*n* = 151)
Intact	50 (33%)
Gel bleed	64 (42%)
Ruptured	37 (25%)

Figure 1 in the supplementary file shows a graphical portrayal of the post-operative implant volume differences, separated per implant state group. Besides a few outliers, it is of interest to note an increase in implant volume in both intact and gel bleeding implants.

In the implant-related outcomes, it was not relevant or statistically possible to analyze the relationships between the ruptured implant variable and the post-operative implant volume, as the post-operative implant volume of ruptured implants logically only had a decrease in volume.

The associations resulting in a post-operative volume decrease or increase are portrayed in Table 4. Capsular contracture resulted in a lower risk of post-operative implant volume increase (OR = 0.15 CI = 0.036–0.59, *P* ≤ 0.05). There appears to be a significant association between bigger implant sizes and post-operative volume differences. As the OR is close to 1.0 for both post-operative implant volume decrease (OR = 1.02 CI = 1.01–1.03, *P* ≤ 0.05), as well as for post-operative implant volume increase (OR = 1.02 CI = 1.02–1.03, *P* ≤ 0.05), this association is not valid enough to have meaningful conclusions. When gel bleed was found as the post-operative implant state, there was a higher chance of developing an increased post-operative implant volume. In univariate analysis, the OR was 4.3 with CI = 1.5–13, (*P* ≤ 0.05), showing an apparent higher risk.

**Table 4:** Univariable logistic regression of outcome post-operative implant volume analyzed per variable (*n* = 152)

	Odds ratio (95% CI*)	*P* value
Age		
Decrease	1.04 (0.98 to 1.1)	0.22
Increase	0.97 (0.93 to 1.0)	0.16
Body mass index		
Decrease	0.92 (0.79 to 1.1)	0.30
Increase	1.01 (0.90 to 1.1)	0.87
Alcohol drinking		
Decrease	0.50 (0.13 to 2.0)	0.32
Increase	0.66 (0.21 to 2.0)	0.46
Years between operations		
Decrease	1.2 (0.79 to 1.7)	0.47
Increase	1.001 (0.77 to 1.3)	0.99
Capsular contracture		
Decrease	0.75 (0.17 to 3.4)	0.71
Increase	0.15 (0.036 to 0.59)	< 0.05
Implant size		
Decrease	1.02 (1.01 to 1.03)	< 0.05
Increase	1.02 (1.02 to 1.03)	< 0.05
Gel bleed		
Decrease	0.25 (0.043 to 1.4)	0.12
Increase	4.3 (1.5 to 13)	< 0.05

*95% CI 95% confidence interval

Table 5 shows the analysis for the outcome of the post-operative implant state. For implants with a post-operative increase in volume, there is a 3.4 higher risk for developing gel bleed, with OR of 3.4 (CI = 1.4–8.3, *P* ≤0.05).

**Table 5:** Univariable logistic regression of outcome post-operative implant state analyzed per variable (n = 152)

	Odds ratio (95% CI*)	*P* value
Age		
Gel bleed	0.97 (0.92 to 1.01)	0.13
Rupture	0.99 (0.95 to 1.03)	0.70
Body mass index		
Gel bleed	1.0 (0.92 to 1.2)	0.56
Rupture	1.1 (0.98 to 1.2)	0.096
Alcohol drinking		
Gel bleed	0.56 (0.18 to 1.7)	0.30
Rupture	0.70 (0.25 to 2.0)	0.49
Years between operations		
Gel bleed	0.93 (0.70 to 1.2)	0.61
Rupture	1.1 (0.77 to 1.5)	0.70
Capsular contracture		
Gel bleed	0.39 (0.073 to 2.1)	0.27
Rupture	1.6 (0.43 to 6.0)	0.49
Implant Size		
Gel bleed	1.01 (1.0 to 1.02)	0.052
Rupture	1.01 (0.999 to 1.01)	0.081
Post-operative implant volume		
Decrease		
Gel bleed	0.65 (0.21 to 2.1)	0.47
Increase		
Gel bleed	3.4 (1.4 to 8.3)	< 0.05

*95% CI 95% confidence interval

The associations related to capsular contracture can be found in Table 6. Post-operative implant shell rupture led to a higher chance of capsular contracture with an OR of 1.8 (CI = 1.01–3.1, *P* = 0.044). An increased post-operative implant volume was related to a lower risk of developing capsular contracture with an OR of 0.23 (CI = 0.081–0.63, *P* ≤ 0.05).

**Table 6:** Univariable logistic regression of outcome capsular contracture analyzed per variable (*n* = 152)

	Odds ratio (95% CI*)	*P* value
Age	1.0 (0.98 to 1.1)	0.21
Body mass index	1.1 (0.82 to 1.4)	0.69
Alcohol drinking	0.53 (0.053 to 5.3)	0.59
Years between operations	0.74 (0.51 to 1.1)	0.11
Implant size	1.0 (0.99 to 1.0)	0.25
Implant state (comparison: intact)		
Gel bleed	0.61 (0.22 to 1.7)	0.33
Rupture	1.8 (1.01 to 3.1)	< 0.05
Post-operative implant volume		
Decrease	0.35 (0.68 to 1.8)	0.22
Increase	0.23 (0.081 to 0.63)	< 0.05

*95% CI 95% confidence interval

## Discussion

The primary aim of this study was to assess the implant dynamics of PIP breast implants and the risk factors related to these implant dynamics. The second purpose of this study was to analyze the rate and predictors for developing capsular contracture in PIP implants. This study shows that the PIP implant shell was highly permeable, leading to an increase or decrease in volume of the implant over the years. Capsular contracture and post-operative implant volume increase had a preventative effect on each other. Gel bleeding related to more implant volume increase and the other way around. Finally, the rupturing of implants led to higher capsular contracture rates.

Capsular contracture poses a protective risk (OR = 0.15, *P* ≤ 0.05) against a post-operative implant volume increase. A possible explanation is that the capsule surrounding the breast implant grows thicker and loses elasticity. Therefore, there is no space for the implant to expand.

Gel bleeding demonstrated a 4.3 (*P* ≤ 0.05) higher risk of increase in post-operative implant volume. Since the definition of gel bleeding is the diffusion of silicone out of the intact implant shell, it is striking that no relation was found between gel bleeding and post-operative implant volume decrease, as one would expect that the exuding of silicone could make the implant lighter. This finding backs the statement that the PIP implant shells were too permeable. Beretta et al. [21] studied the (physico)chemical properties of PIP silicone, comparing it with silicone from a McGhan implant model. They concluded that the silicone used by PIP was low-cohesive because of the method used to manufacture the chemicals, where polymers were not properly cross-linked. They proved that this silicone gel could adsorb substances like cholesterol from surrounding tissue, which could in turn lead to a lower viscosity and eventually higher risk of rupture. In a subsequent study, Beretta et al. [22] analyzed the biochemical structure of accumulated fluid around PIP implants. They showed that low and high molecular weight silicone can diffuse not only out of the implant shell but also out of the capsule formed around the implant and into the body. They also managed to illustrate that, besides cholesterol, other endogenous substances like uric acid, globulins, and albumin could enter the implant shell. They hypothesized that this diffusion occurs because of the osmotic power present and might be responsible for the increase in volume despite the gel bleed. Swarts et al. [5] also reported a higher risk of implant rupture due to lack of impermeability. PIP implant shells were proven to have been manufactured with inconstant degrees of thickness, sharp edges, and miniature holes in the hollow parts of textured shells. Additionally, the anti-bleeding barrier layer that is customarily fabricated in modern implants appeared to not have been included in the PIP implant model since 2007 [5].

The previous finding is strengthened, since a post-operative implant volume increase seems to lead to a 3.4 (*P* ≤ 0.05) higher risk of developing gel bleeding. If we hypothesize that the implant volume increased before the start of gel bleeding, one could argue that a larger content in the implant shell leads to a higher mechanical pressure and eventually leakage of silicone into the surrounding tissue. In the case that this process does not occur in that strict order, it is still very apparent that the PIP implant shell worked as an unsuitable protective layer that lets fluid through in both directions.

Regarding risks for developing capsular contracture, ruptured implants at explantation were associated with an OR of 1.8 (*P* ≤ 0.05). Studies by Feng et al. [23] and Holmich et al. [24] found similar risks. The large amount of silicone that is released after rupture could be a stimulus of the process of contracture. Alternatively, it could also be the effect of the mechanical changes of the ruptured implant that in turn cause friction with the surrounding tissue, leading to the formation of fibrous tissue. The opposite is also possible, namely, that capsular contracture leads to the rupturing of implants because of a higher mechanical pressure on them. Our cohort showed a rupture rate of 25%, which is comparable with the literature that reported a wide range of 3.9 to 31.6% [25–32]. Breasts that developed capsular contracture contained ruptured implants in 33% of the cases, which was not increased in comparison to intact (39%) and gel bleeding (22%) implants.

Additionally, post-operative implant volume increase seems to have a protective effect (OR = 0.23, *P* ≤ 0.05) against capsular contracture. Again, the capsular contracture surrounding the implant could prevent any possible expansion of the implant, because the contracted capsules tend to tighten around it. Since it is not possible to determine which phenomenon develops first, this outcome can, in the least, be interpreted as there being a clear association between the two variables.

The results of our study point towards the fact that PIP implant shells indeed lacked adequate impermeability and thus were prone to develop a change in post-operative implant volumes. This, in combination with low-quality silicone gel, provides a high risk for rupture and, thus, a higher probability for developing capsular contracture.

In evaluation of our study, we acknowledge that our study was conducted on a targeted group of patients following the PIP incident. What can be perceived as a limitation is the fact that these implants have been banned from use. However, due to the consequences of the PIP breast implant scandal and the high explantation rate, there have been many studies published on different aspects of this particular breast implant. This provides us with the opportunity to relate the different aspects to factors that influence shell rupture, gel bleeding, and capsular contracture, all of which are complications regularly occurring in patients with new models of breast implants. If we can compare these factors in the PIP implants, and compare the identified factors with breast implants that are currently being used, we will be able to better understand the causes for these complications. One major limitation that this scenario offers is the lack of a control group. Comparison of our data with other brands of implants could have provided a more clinically relevant outcome for our analyses. Furthermore, there appeared to be three different batches of silicone used in the implants throughout the years. The PIP silicone used to measure density was of the PIP2 batch, while the PIP implants of our cohort were predominantly from the PIP1 or NUSIL batch [33]. Although this density was the closest way of determining the post-operative volume, it might not be completely applicable to every PIP implant model used, as there is no traceable record of the component of different batches.

With a cohort of this size and the retrospective design of this study, a conclusion can be limited due to a high chance of bias, loss of data, or confounding factors. Missing data was considered random, since the investigators who collected the data were independent from the involved physicians in the patient care and only used data accessible in patient files. Also, this led to different sizes of variable groups, which made multivariable statistical analysis less reliable. A considerable advantage of our group was the follow-up time of 11 ± 2.1 years, which is a clinically satisfactory amount of time of implantation to observe the impact implants can have on the body and vice-versa. This study set-up was, to our knowledge, the first of its kind, measuring the post-operative implant volume difference in a cohort of this size and relating it to the post-operative implant state and development of capsular contracture.

In conclusion, we illustrated that the elastomer shells of PIP implants were functioning deficiently, since our cohort has shown that volume increase as well as decrease widely occurred. Consequently, there seems to be a correlation between gel bleeding implants and the increasing of the post-operative implant volume. In contrast, capsular contracture could have a protective effect against post-operative implant volume increase, and a post-operative implant volume increase could also provide a protective influence in developing capsular contracture. Additionally, implant ruptures led to a higher probability for capsular contracture. We unquestionably encourage more research on the dynamics of breast implants, particularly with implant models that are currently on the market. We also urge official health institutions to implement stricter guidelines following Hazard Analysis and Critical Control Points (HACCP) to ensure a higher quality of breast implants and thus better patient safety.

## Electronic supplementary material

ESM 1 (DOCX 23 kb)
