# Supplementary material for: Poly Implant Prothèse silicone breast implants: implant dynamics and capsular contracture
Source: Eur J Plast Surg. 2018 Jun 1;41(5):563–70. doi: 10.1007/s00238-018-1427-y (PMC6153863; doi:10.1007/s00238-018-1427-y)
Supplement: Supplementary file 1 — (DOCX 23 kb) [file 238_2018_1427_MOESM1_ESM.docx]

**Supplementary file**

**Figure 1: The boxplots portray the post-operative implant volume differences per patient, displayed per implant state. The x-axis is the patient number and the y-axis is the volume difference in milliliters.**
